# Qualitative expert evaluation of an educational intervention outline aimed at developing a shared understanding of cross-border healthcare

**DOI:** 10.3205/zma001672

**Published:** 2024-04-15

**Authors:** Juliëtte A. Beuken, Mara E.J. Bouwmans, Diana H.J.M. Dolmans, Michael F.M. Hoven, Daniëlle M.L. Verstegen

**Affiliations:** 1 Maastricht University, School of Health Professions Education, Department of Educational Development and Research, Maastricht, The Netherlands

**Keywords:** design-based research, interprofessional education, team learning, cross-border health care

## Abstract

**Objectives:**

Although cross-border healthcare benefits many patients and healthcare professionals, it also poses challenges. To develop a shared understanding of these opportunities and challenges among healthcare professionals, we designed an educational intervention outline and invited experts in healthcare and education to evaluate it. The proposed intervention was based on theoretical principles of authentic, team, and reflective learning.

**Methods:**

Experts (N=11) received a paper outline of the intervention, which was subsequently discussed in individual, semi-structured interviews.

**Results:**

Based on a thematic analysis of the interviews, we identified 4 themes: 1) using the experience you have, 2) learning with the people you work with, 3) taking the time to reflect on the past and future, and 4) adapting the intervention to its context.

**Conclusion:**

According to the experts, the proposed intervention and its three underlying principles can enhance a shared understanding of cross-border healthcare. To unlock its full potential, however, they suggested adjusting the application of learning principles to its specific context. By situating learning in landscapes of practice, the intervention could contribute to the continuous development of cross-border healthcare.

## Zusammenfassung

**Zielsetzungen:**

Obwohl die grenzüberschreitende Gesundheitsversorgung für viele Patienten und Angehörige der Gesundheitsberufe von Vorteil ist, bringt sie auch Herausforderungen mit sich. Um ein gemeinsames Verständnis dieser Chancen und Herausforderungen unter den Fachkräften des Gesundheitswesens zu entwickeln, haben wir eine pädagogische Intervention entworfen und Experten aus dem Gesundheits- und Bildungswesen eingeladen, diese zu bewerten. Die vorgeschlagene Intervention basierte auf den theoretischen Grundsätzen des authentischen, teamorientierten und reflektierenden Lernens.

**Methoden:**

Die Experten (N=11) erhielten einen Entwurf der Intervention in Papierform, der anschließend in individuellen, halbstrukturierten Interviews diskutiert wurde.

**Ergebnisse:**

Auf der Grundlage einer thematischen Analyse der Interviews identifizierten wir vier Themen: 1) Nutzung der eigenen Erfahrung, 2) Lernen mit den Menschen, mit denen man zusammenarbeitet, 3) sich Zeit nehmen, um über die Vergangenheit und die Zukunft zu reflektieren, und 4) Anpassung der Intervention an den jeweiligen Kontext.

**Schlussfolgerung:**

Nach Ansicht der Experten können die vorgeschlagene Intervention und die ihr zugrunde liegenden drei Prinzipien ein gemeinsames Verständnis der grenzüberschreitenden Gesundheitsversorgung fördern. Um ihr volles Potenzial auszuschöpfen, schlagen sie jedoch vor, die Anwendung der Lernprinzipien an den spezifischen Kontext anzupassen. Durch die Verortung des Lernens in *Landscapes of Practice* könnte die Intervention zur kontinuierlichen Entwicklung der grenzüberschreitenden Gesundheitsversorgung beitragen.

## 1. Introduction

Both patients and healthcare professionals (HCPs) could profit from cross-border healthcare, thus improving regional healthcare [1]. In European border regions many patients and professionals cross country borders daily. It makes sense for a person in need of acute or planned care to go to the nearest hospital, which might be just across the border (e.g., after a cycling accident or for complex surgery). 

**Figure 1:**
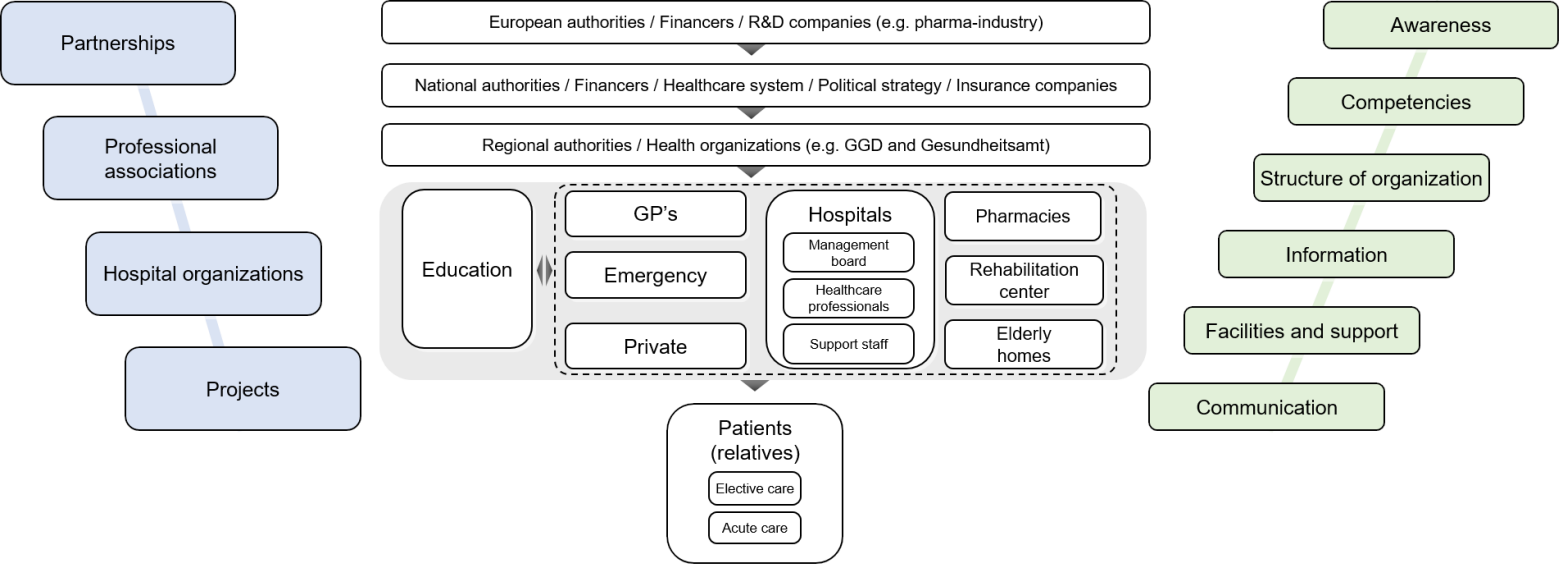
The complexity of the system

Although a decrease in travel time and the costs of specialized or acute treatment are great advantages, the challenges HCPs face are bigger than those in “regular” healthcare. Language barriers, cultural differences, unfamiliarity with other teams, and variations between healthcare systems [2], [3], [4] are but a few of the challenges that can contribute to a loss of information, in turn posing risks to patient safety [5], [6]. The complexity of this system is illustrated in figure 1 (Fig. 1). Hence, the challenges HCPs face in cross-border healthcare is a topic that needs addressing.

Standardized protocols may go a long way in facilitating the handover of information [7], [8] and policy development may provide relief in organizational matters [9]. Other challenges, like differences in tasks, education, and hierarchy, call for a shared understanding of healthcare variety between countries [10]. This shared understanding, and the associated challenges and opportunities, resonate with the concept of shared mental models [11]. Shared mental models are knowledge structures held by members of a team that enable them to gain a collective understanding of a task and the expectations attached to it. With a shared mental model, members can coordinate their actions accordingly, adapting their behavior to the demands of the task and their fellow team members [11]. In certain situations, shared mental models allow team members to gauge expectations and adapt accordingly.

Yet, it takes time and effort to establish this shared understanding of cross-border healthcare challenges and opportunities. An earlier study concluded that discussions between the collaborating HCPs are essential to inform individual mental models and facilitate the development of a shared mental model [12]. Although such discussions may occur naturally when HCPs regularly come together, teams of HCPs in cross-border collaborations meet only sporadically or not at all (e.g., when information is transferred solely on paper). This hinders the natural development of shared mental models. In such cases, education may be the answer to aid the development of a shared understanding.

Current cross-border healthcare education is limited [2] and does not meet the design strategies suggested to develop this shared understanding. Rather than using a more comprehensive educational approach to enhance the transfer of learning to the workplace [13], [14], [15], it addresses single topics such as specific checklists or systems [2]. We identified three design principles that are instrumental in developing a shared understanding of cross-border healthcare, which should be central to cross-border healthcare education: authentic learning, team learning, and reflective learning. The first principle, authentic learning, refers to learning with authentic problems and tasks to stimulate the transfer of learning to practice. It allows participants to learn from and for situations in which their knowledge is to be used [16], [17]. The second principle, team learning, entails that participants share individual perspectives (e.g., on a problem or situation), negotiate differences using arguments and clarifications, and collaboratively construct a shared perspective [18]. The third principle, reflective learning, refers to participants critically questioning their own ideas. Reflection helps participants to see the boundaries of their own perspective and recognize missing information [19], [20]. Moments of reflection in education will help participants to understand, broaden, or change their own views on cross-border healthcare. An intervention that is based on these principles may support HCPs in developing a shared understanding of cross-border healthcare. Therefore, we designed an outline of such an educational intervention for HCPs with the aim to foster a shared understanding of cross-border healthcare and the challenges and opportunities associated with it. The intervention was designed for ten to fifteen HCPs from interdisciplinary teams collaborating or planning to collaborate across the border. First, participants prepare an example of cross-border healthcare from their own practice. Second, in an interactive session, participants discuss challenges and opportunities in the examples they prepared. They then reflect on an overview of cross-border healthcare (see figure 1 (Fig. 1)) and further discuss challenges in their respective collaboration. Finally, participants discuss possible strategies to improve their collaboration. Third, after the interactive session, participants reflect on what they have learned in the form of take-home messages. The intended learning outcomes of this intervention are threefold: To become aware of

1. challenges and opportunities of cross-border healthcare, and
2. construct strategies to improve collaboration.

The elaborate outline of the intervention is provided in attachment 1 .

In the current study, we evaluated the proposed intervention with experts in healthcare and education. Our research question was: *How do experts in healthcare and education evaluate an educational intervention outline with elements of authentic, team, and reflective learning designed to stimulate a shared understanding of cross-border healthcare challenges and opportunities among HCPs?*

## 2. Methods

To evaluate the educational intervention outline described in the Introduction, we adopted an educational design research (EDR) approach. The focus of this approach is to use appropriate design principles – insights from educational theory and practice – when designing education [21], [22] with the dual aim of advancing education both in theory and in practice. We invited experts from the field of healthcare and education to evaluate the outline in semi-structured interviews.

### 2.1. Setting

The study took place in the Meuse-Rhine Euregion, where the borders of Belgium, Germany, and the Netherlands meet. Ever since its establishment in 1976, the region has been a pioneer of cross-border healthcare [1]. Next to a cross-border network for acute care, several departments in the regional hospitals have collaboration agreements to enable patients and HCPs to cross borders for healthcare.

### 2.2. Respondents

Respondents were experts from healthcare and education. They qualified as “healthcare experts” when they had over one year of experience in providing and/or organizing cross-border healthcare (e.g., as a nurse or a policy adviser in a hospital in the border region) and as “education experts” when they had over one year of experience in health professions education. Considering these inclusion criteria, we recruited experts from our own network, striving for a variety in professional and national backgrounds (convenience and purpose sampling methods). We informed experts about the study procedure and invited them to participate by email. We approached 12 people, 11 of which were included in the study. Respondent characteristics are provided in the Results section. 

### 2.3. Data collection

Before the interviews, respondents received a document outlining the aim and design of the intervention. The outline informed experts of the intervention’s goal, target group, intended learning outcomes, and the components to achieve these. The elaborate outline of the intervention is provided in attachment 1 . In interviews of approximately 60 minutes, we asked respondents about their general impression of this outline, before asking specific questions about the intervention’s design and use of learning principles (e.g., *“Do you think authentic learning is integrated well into the design? Explain why [or why not] and give an example”*). At the end, we asked respondents for suggestions to improve the intervention outline. The interview guide was slightly adapted after a test interview and is provided in attachment 2 . Either a senior researcher or the first author (JB) conducted the interviews, with the last author (DV) or the first author (JB) being present as observer. Interviews were audio-recorded and transcribed by either an independent transcription company or the first author (JB).

### 2.4. Data analysis

Data were analyzed thematically using Atlas.ti, version 9, and following Braun and Clarke [23]. Two researchers (JB and DV) analyzed six interviews using the learning principles and intended learning outcomes as sensitizing concepts. After a discussion about additional themes that arose, three researchers (JB, DV, and MB) coded all interviews using a coding scheme. The outcomes of this thematic analysis constituted the first three themes described in the Results section. In numerous discussions about these results, we conceptualized a critical reflection on the interviews, which resulted in a fourth theme.

### 2.5. Researcher backgrounds

Our professional backgrounds influenced our data collection and analysis. JB is a qualitatively trained researcher with a degree in health sciences. MB is a quantitatively trained researcher with a degree in Psychology. Together, they have interviewed over 50 professionals and patients about their healthcare experiences in border regions for previous studies. DD is an educational scientist who has researched small-group teaching in medical education from a cognitive, social, and, notably, a student and supervisor perspective. MH is a researcher with a degree in psychology. His research focuses on team learning behavior in emergency contexts. Finally, DV is an educational and cognitive scientist who has conducted research in the field of instructional design and international education.

### 2.6. Ethics

This study was considered and approved by the Faculty of Health, Medicine and Life Sciences Research Ethics Committee (FHML-REC/2019/043/Amendment 1).

## 3. Results

From October to December 2020, we interviewed experts in healthcare (N=7) and education (N=4). Three of the experts in healthcare were physicians, two were nurses, and two were involved in policy development. Two respondents were from Germany, two from Belgium, and the remaining seven participants were from the Netherlands. Respondent characteristics can be found in table 1 (Tab. 1). Interviews were conducted in Dutch or English and lasted 50 minutes on average, ranging from 38 to 63 minutes. Given our aim was to evaluate an intervention outline with healthcare and educational experts, and the respondents in this sample provided agreeing, diverging and contrasting views on the outline, we believe this respondent sample was substantial and significant (enough) as a study basis [24]. 

We describe the four themes that we identified in our analysis. The first three themes reflect respondents’ ideas about the authentic, team, and reflective learning principles and how they were applied in the intervention:

1. Using the experience you have,
2. Learning with the people you work with, and
3. Taking the time to reflect on the past and future.

The fourth theme denotes that the intervention should be adapted to context-specific aspects of cross-border collaboration:

1. Adapting the intervention to its context.

### 3.1. Theme 1: Using the experience you have

According to respondents, authentic learning in this intervention contributes to a shared understanding. As future participants would already have some experience of cross-border healthcare, they even considered authentic learning indispensable to the intervention: It would motivate participants to get actively involved in the intervention. Respondent 3 (educational expert) mentioned:

*Because it then arises from a question that really lives, … at least those people are dealing with it at that moment, so it is not some far-off thing, but it has something to do directly with their day-to-day work and I think that makes you more motivated.*

Next to this, the experts also considered participants learning *for* their own setting, or translating what they learned into explicit actions, as an important part of the intervention: 

*“Imagine that you have that take-home message, that you ask them to explicitly link it to those strategies for improving …, and then also get started with it as a team ….” *(Respondent 3, educational expert)

However, respondents agreed that the use of participants’ *own *experiences in the intervention should be counterpoised by shared experiences as well. As respondent 5 (healthcare expert) said:

*They all have their own agenda and their own things. Anyway, on all sides it is now time for change. I think so. … Ensure that there is a mission that is supported, …. Not just, ‘oh I benefit from it,’ but just being really supported by … the group.*

### 3.2. Theme 2: Learning with the people you work with 

The intervention was designed for people from different professions and cultures who collaborate across borders. Indeed, respondents saw existing collaborations as the cornerstone of the intervention: 

*“… They have already found each other, and they are already doing business together and they just want to take that to a higher level.” *(Respondent 10, healthcare expert) 

For the intervention to be relevant, they felt that participants should learn together with the people they also work with. As respondent 6 (healthcare expert) said: 

*“I think it makes little sense to do such an [intervention] with people who do not work together, or about a completely different case or something. … That is completely irrelevant.”*

Respondents also pointed to the complexities of different professions and cultures that come together in the intervention. Respondent 5 (healthcare expert), a physician, said it is *“very difficult to get physicians to work well in a group …”*, and others mentioned that cultural differences could affect participants’ ability to learn together. However necessary for team learning, open discussions could be challenging in interprofessional and intercultural groups. Respondent 9 (healthcare expert) pointed out that 

*“The hierarchy in a hospital is different in country A than in other countries. … I also really dare to give feedback to a supervisor if I disagree with something. What I notice is that this is not so easy in country B. … The question is: How open will they be during such [an intervention]?”*

At the same time, respondents acknowledged that one could not predict how the group dynamics affect learning in the intervention. They therefore emphasized moderators’ role in facilitating a safe learning climate so that participants felt safe to voice their own ideas. Moderators could do this by making this safe learning climate explicit:

*By emphasizing from the outset that this contribution from everyone together contributes to better care or better patient safety, you can, say, achieve a safe climate. … And you must first create such a situation, but then really name it …. *(Respondent 4, education expert) 

### 3.3. Theme 3: Taking the time to reflect on the past and future

Respondents felt rather ambivalent about reflection assignments: While on the one hand they considered these as mere administrative exercises, on the other they recognized their use in connecting the intervention to practice. If included, they should meet three criteria. First, reflection should help participants make translations from the intervention to their own situation. As respondent 1 (education expert) said: 

*“You cannot just ask someone something and expect that they immediately open a drawer in their brain and share a memory. … They need help with that, I think.”*

Second, it should be a group exercise, aimed at the next steps of cross-border collaboration: 

*“… You can then discuss things, and learning objectives, or new challenges, and also make a plan of action together.” *(Respondent 9, healthcare expert) 

And finally, the reflection exercise should be aimed at future plans: 

*“For me, reflection is never a goal in itself. For me, reflection is always a means to move forward.”* (Respondent 7, education expert)

### 3.4. Theme 4: Adapting the intervention to its context

Respondents’ ideas about what the intervention should look like in terms of its target group, goal, and rollout differed depending on their own backgrounds and experiences of cross-border healthcare. These differences also arose from the various contexts they had in mind. Respondent 11 (healthcare expert), for instance, thought of cross-border healthcare as something that was yet to be organized and therefore suggested that the intervention target managerial staff: 

*“People with … power. You know, a good vision of what is possible or what is not possible ….”*

This did not include nurses, paramedics, or physicians. Another respondent, however, felt the intervention would be more appropriate for nurses and administrative staff who were involved in the collaboration but did not usually meet: 

*“I think it is very good, especially for people who mainly [speak] by phone, and by email, to see a face once, and to sit in an [intervention] together. That can really help.” *(Respondent 9, healthcare expert) 

With respect to the intervention’s goal and concrete implementation, one of the respondents suggested that the intervention could provide HCPs who were in an orientation phase the opportunity to talk about 

*“something you experienced, what went well, what went wrong”* (Respondent 2, healthcare expert). 

Respondent 8 (healthcare expert), who was considering the same stage of implementation, added: 

*“Then it is more about letting things soak [in] and getting people to think about partnerships from their own practice”*. 

Yet other participants saw the intervention’s rollout when people were *“already in a trajectory”* (Respondent 2, healthcare expert), and therefore opined that goals should be more specific. In such cases, 

*“the best thing would be for the team to say: We would like to do this [intervention] because we see that something is going wrong here ….”* (Respondent 4, education expert).

## 4. Discussion

The experts interviewed in our study confirmed that an educational intervention based on authentic, team, and reflective learning can be instrumental in developing a shared understanding of cross-border healthcare among involved HCPs. They concluded that HCPs who are – or are planning to be – involved in cross-border healthcare should be stimulated to use their own experiences (theme 1) to learn with those they collaborate with (theme 2). Reflection helps to discuss experiences and opportunities for improvement (theme 3). While these learning principles are generally helpful, the way in which they take shape in an intervention should vary according to the situation (theme 4). When physicians, nurses, and paramedics are involved in cross-border collaboration, they should all participate in the intervention. However, the group composition will affect how participants learn together. In early-stage collaborations, the intervention’s focus should be on future rather than existing challenges, which calls for different reflections. In established collaborations, the intervention could focus on specific barriers or problems. Hence, during its implementation, the intervention should be adapted to context-specific factors (such as aims and form of collaboration, involved professions, languages spoken, etc.).

Making education in context means that situation specifics determine how we should translate learning principles into educational practice. Cianciolo and Regehr [25] pointed out that we should pay attention to the context in which an intervention is implemented. Contextual factors (e.g., how participants are already collaborating or cultural differences among them) influence the intervention’s structure and implementation in practice (e.g., which problems to address, who participates in the intervention, or what expertise facilitators should have). The learning principles should offer guidance in this process of contextualization [25]. In the case of authentic learning, for instance, the challenges being discussed will differ across contexts, without compromising the principle of authenticity as an essential part of the intervention. Conscientious adaptation of the design allows tuning to the context, while preserving its essence. It is helpful to include participants who know what is relevant in their situation, in the preparation of the intervention. This will strengthen the impact of the intervention in different stages of cross-border healthcare development.

This study emphasizes the importance of shared learning. When participants feel safe to share and discuss their experiences (i.e., if the learning climate is safe) and if they have a sense of interdependence among them, they learn more easily [17]. As respondents mentioned, participants should recognize the value of others and the possibilities to learn from them. In order to learn as a team, they have to recognize the common goal to which they all contribute (i.e., patient care). To support this process, they should engage in activities that foster such kind of team development [18]. Group facilitators should help participants to take control of what and how they learn together [26]. The intervention can ultimately stimulate the formation of a landscape of practice [27], in which HCPs from different communities of practice are aware of each other’s value in cross-border healthcare. Landscapes of practice will facilitate a more dynamic and continuous way of shared learning, which grows as cross-border collaborations develop.

This study has several strengths. Our mixed group of participants – healthcare and education experts – provided a variety of perspectives. Especially respondents with experience in cross-border healthcare helped reveal the importance of context. Furthermore, we applied a theory-informed approach to designing and evaluating our educational intervention outline, while carrying out the evaluation in close collaboration with various stakeholders. We are also aware of limitations. Although we included respondents from three countries, we are aware of cultural imbalance in the study, as most of the respondents (64%) and authors (80%) were Dutch. Notably, 100% of education expert respondents were Dutch, meaning that diversity in education, namely from Belgium and German perspectives, was not represented. Finally, respondents evaluated a paper outline of the intervention. It is challenging to envision a four-hour intervention and its potential follow-up meetings based on one-dimensional information.

We consider several practical implications, for educational activities on cross-border healthcare in general, and specifically for our intervention. We should not present (all) educational interventions for healthcare practice as isolated initiatives. The educational character of our intervention evokes the expectation that there is one moment in time at which all issues are resolved. Rather, educational interventions should be part of a process of learning to recognize and discuss new issues as they arise. Thus, we might repeat educational interventions, so they become part of a learning continuum (of *Communities of Practice* and *Landscapes of Practice*). In establishing a learning continuum, educational interventions need to take shape within context, meaning that with every time the intervention is ‘repeated’, its goals, activities and target groups are reconsidered. For our intervention, this meant embedding a preparatory phase in which we gain further insight into the cross-border healthcare situation in which the intervention is implemented, i.e. by speaking to several HCPs involved in the collaboration beforehand. We can identify tensions in the learning principles as imagined and contextual limits, and consider alternatives. For example, collaborative learning is limited if participants are not fluent in the same language. Depending on situational factors, we could then consider a translator, asking trainers who speak different languages, or providing more preparation material. 

Future research should test the intervention and explore how the educational design principles interact with the context in which they are implemented. In such an implementation study, special attention should be paid to the impact of cultural and professional differences on learning principles. We suggest implementing the intervention in different situations, with a variety in context and stage of collaboration. Moreover, implementation research could provide insight into how shared understanding of cross-border care among HCPs contributes to concrete actions, like (revisions of) collaboration agreements or opportunities for joint skill training. Finally, though this study focused specifically on cross-border collaborations in healthcare, the outcomes might be transferable to contexts outside of healthcare. We see potential to investigate cross-border learning in other contexts where people from different countries collaborate closely. 

The design of cross-border healthcare education should be based on both practical needs and theoretical insights. Authentic, team, and reflective learning are useful to support a shared understanding of cross-border healthcare. However, what learning principles do depends not only on how they applied in educational design, but also on the context in which they are applied. Educational design based on theory and practice cannot be marked off as ready for implementation: It should be made in context.

## 5. Conclusion

We summarize the conclusions of this study in three key points.

1. An intervention with characteristics of contextual, team and reflective learning has potential to enhance HPCs awareness of cross-border healthcare challenges and opportunities.
2. Educational interventions for cross-border healthcare could be repeated, and become part of a process of learning to recognize and discuss new issues as they arise in collaborations.
3. Educational interventions for cross-border healthcare should take shape within context; goals, activities and target groups should be tailored to the participants’ context.

## Funding

This research was funded by the European Regional Development Fund (ERDF) and regional provinces (co-financing) as part of the Interreg V-A SafePAT project (EMR90).

## Authors’ ORCIDs

- Juliëtte Beuken: [0000-0002-5431-9418]
- Mara Bouwmans: [0000-0003-4930-5051]
- Diana Dolmans: [0000-0002-4802-1156]
- Michael Hoven: [0000-0002-1050-2683]
- Daniëlle Verstegen: [0000-0001-6811-175X]

## Competing interests

The authors declare that they have no competing interests. 

## Supplementary Material

Intervention outline

Interview guide

## Figures and Tables

**Table 1:**
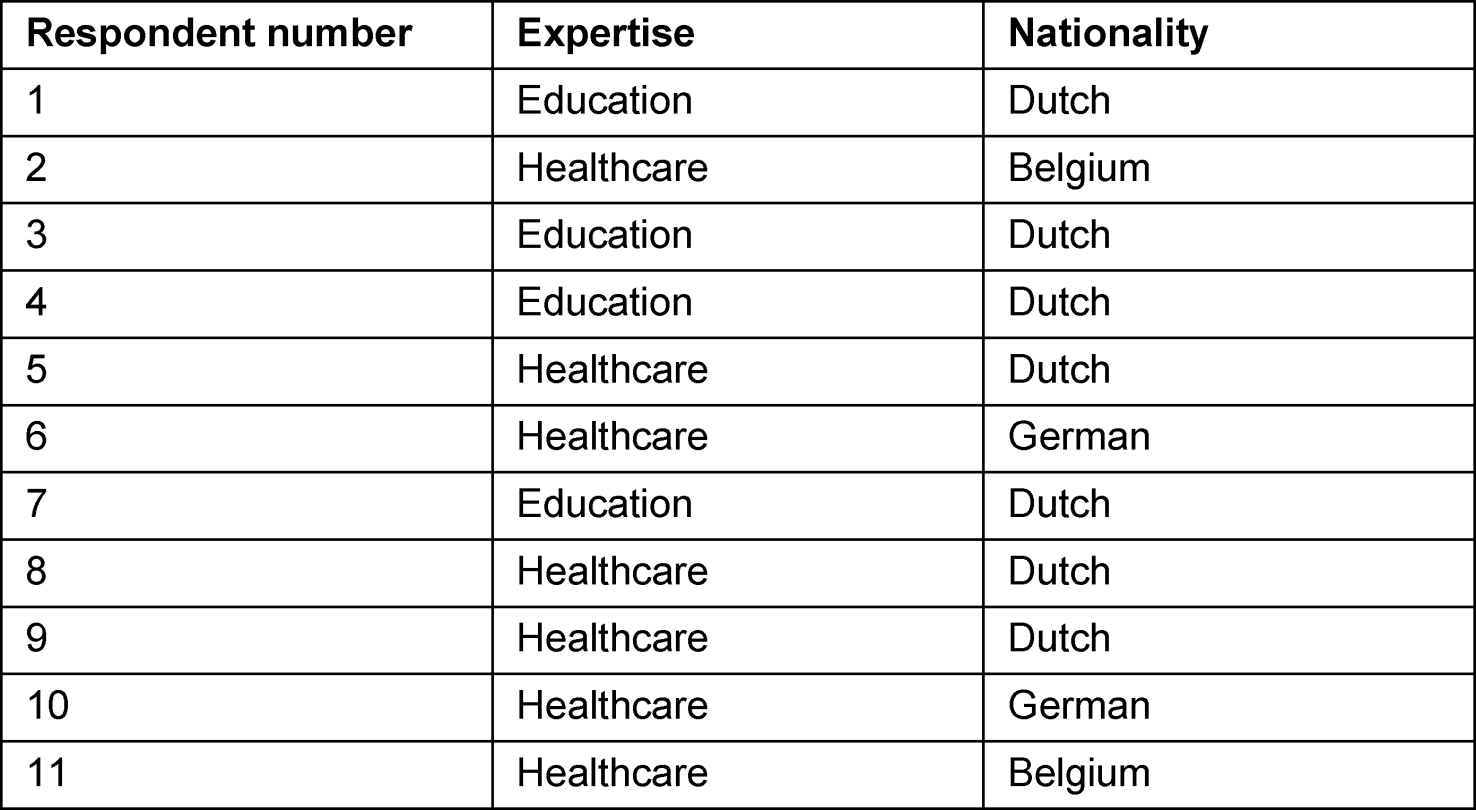
Respondent characteristics
