# Supplementary material for: Qualitative expert evaluation of an educational intervention outline aimed at developing a shared understanding of cross-border healthcare
Source: GMS J Med Educ. 2024 Apr 15;41(2):Doc17. doi: 10.3205/zma001672 (PMC11106574; doi:10.3205/zma001672)
Supplement: Intervention outline [file JME-41-17-s-001.pdf]

Attachment 1: Intervention outline

Crossing Borders in Cross-border Healthcare
Intervention outline

INTRODUCTION

More and more, patients cross borders, for example when hospitals directly across the border provide urgent or specialized care. Cross-border healthcare can be beneficial for patients, but also comes with patient safety risks. Recent research has revealed that healthcare professionals involved in cross-border healthcare experience challenges with, for example, information transfer, language barriers, differences in health care systems, education and culture.

Healthcare professionals are sometimes unaware of the cross-border differences underlying these challenges. They often say that they do not know their international colleagues very well and that they do not know much about other healthcare systems. As a result, healthcare professionals in cross-border healthcare might not know what to expect from one another in collaborations.

The intervention titled *Crossing Borders in Cross-border Healthcare* aims to raise awareness about the opportunities and challenges of existing cross-border healthcare collaborations. The intervention creates an opportunity for healthcare professionals to learn from, with and about each other. Using authentic examples helps them gain insight in why and how they are relevant to each other, and how they can align their activities to improve cross-border healthcare.

Target group

The intervention is designed for groups of healthcare professionals who already collaborate with each other across borders. This concerns for example departments with existing partnerships for specialized treatment, or emergency departments frequently visited by foreign ambulances. The intervention is provided upon request (e.g. by management of the department, or international policy advisors). Healthcare professionals directly involved in the collaboration (physicians, nurses, residents and allied healthcare professionals, such as paramedics and administrators) are invited to participate. The intervention is provided to a group of ten to fifteen healthcare professionals from the different countries involved in the collaboration.

Intended learning outcomes

The intervention has the following intended learning outcomes.
These are also communicated to the participants.

- Healthcare professionals are aware of the opportunities of the cross-border healthcare collaboration.
- Healthcare professionals are aware of the challenges of the cross-border healthcare collaboration.
- Healthcare professionals know how to utilize the opportunities and overcome the challenges.

To achieve these intended learning outcomes, it is important that healthcare professionals get the opportunity to get to know each other and discuss their collaboration. The intended learning outcomes ultimately facilitate a so-called landscape of practice, in which healthcare professionals from different countries have shared ideas about the purpose, responsibilities and goals of their collaboration.

DESIGN OF THE INTERVENTION

The intervention exists of three parts: 1) a preparatory assignment, 2) a four-hour interactive session, and 3) a reflection assignment. Description of the design is provided below.

The default language of the intervention is English. Depending on participants' preferences, the language in which the intervention is provided, can be discussed.

The interactive session is provided by trainers from the participating countries. At least one trainer should have experience with cross-border healthcare.

1) Preparatory assignment

Format Individual, on paper

Description A week prior to the intervention, participants are asked to provide examples of their own experience with cross-border healthcare. Questions participants are asked to answer are: "Describe an example of cross-border healthcare you were recently involved in. What went well? What was challenging?"

The assignment provides input for the interactive intervention.

Time *30 minutes*

2) Interactive session

Format Group, in person

Description The interactive sessions start with participants discussing the examples of cross-border healthcare from the preparatory assignments. They discuss both challenges and opportunities in these examples. Thereafter, trainers provide a schematic overview (figure 1) and examples of cross-border healthcare in the region (resulting from previous research). Healthcare professionals discuss to what extent they recognize this overview, or how they think it differs from their own perception. Then, healthcare professionals discuss what challenges they experience in cross-border healthcare, and what they believe is the cause of these challenges. In this task, it is important that healthcare professionals react to each other, and learn from, with and about each other's experiences and feel safe to discuss disagreements. Next, healthcare professionals brainstorm about strategies to improve cross-border healthcare, based on previously discussed examples. Participants discuss what is needed to improve cross-border healthcare in their setting and collectively formulate strategies to strengthen their cross-border collaboration in practice.

Time 180 minutes

3) Reflection assignment

Format Individual and group, on paper

Description A week after the intervention, participants are asked to reflect briefly on the intervention and list their main take-home messages. These take-home messages are summarized by the trainers and shared with all participants. The summary serves as a starting point for further actions by the participants themselves.

Time 30 minutes

Figure 1 – Schematic overview of cross-border healthcare

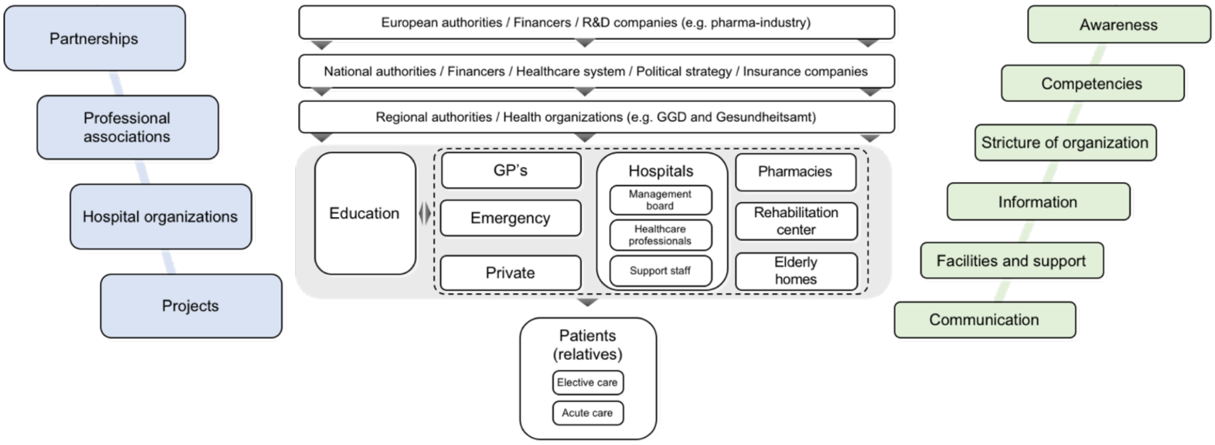

LEARNING PRINCIPLES

We used three principles in the design of the intervention. These three learning principles are integrated in different parts of the intervention.

1) Authentic learning	Participants are triggered to learn from professionally relevant or meaningful real-life cases they experience in their own practice or experiences from others (e.g. by using their own examples or other examples of cases), to enhance transfer of learning to practice.
2) Team learning	Participants share and discuss individual perceptions (e.g. of a problem or situation), negotiate differences in perceptions using arguments and clarifications, and collaboratively construct a shared perception. This should encourage participants to learn from, with and about each other to enhance future cross border collaboration.
3) Reflective learning	Participants are triggered to question their own judgements and recognize missing information, which ultimately leads to a deeper understanding. They are stimulated to reflect on how they collaborate, and own their own role in the collaboration.

Attachment 1 to Beuken JA, Bouwmans ME, Dolmas DH, Hoven MF, Verstegen DM. *Qualitative expert evaluation of an educational intervention outline aimed at developing a shared understanding of cross-border healthcare.* GMS J Med Educ. 2024;41(2):Doc17. DOI: 10.3205/zma001672
