# Supplementary material for: Qualitative expert evaluation of an educational intervention outline aimed at developing a shared understanding of cross-border healthcare
Source: GMS J Med Educ. 2024 Apr 15;41(2):Doc17. doi: 10.3205/zma001672 (PMC11106574; doi:10.3205/zma001672)
Supplement: Interview guide [file JME-41-17-s-002.pdf]

Attachment 2: Interview guide

INTRODUCTORY QUESTIONS

1. **What is your first impression of the workshop?**

The workshop has three parts that are supposed to build upon each other (preparatory assignment, interactive session and reflection assignment).

2. **What are your thoughts on the general set-up of the workshop?**
   a. *What do you like about the design and why? What do you not like and why? Give examples.*
3. **What are your thoughts on the intended learning outcomes?**
   a. *To what extent the intended outcome fit the target group? Explain why and give an example.*
   b. *To what extent are the intended learning outcome relevant to the target group and/or improving cross-border care? Explain why and give an example.*

LEARNING PRINCIPLES AND LEARNING OUTCOMES

As you have read in the description, the design of the workshop is based on three principles: authentic learning (learning based on professionally relevant problems), team learning, reflective learning and has particular intended learning outcomes in mind and a specific target group of participants.

4. **Do you think authentic learning (learning based on professionally relevant problems) is integrated well in the design?**
   a. *Explain why (not) and give an example.*
   b. *How does it enhance transfer of learning to practice?*
5. **Do you think team learning is integrated well in the design?**
   a. *Explain why (not) and give an example.*
   b. *How does it enhance team learning in practice?*
6. **Do you think reflective learning is integrated well in the design?**
   a. *Explain why (not) and give an example.*
   b. *How does reflective learning enhance transfer of learning to practice?*
7. **Is there any other principle that might deserve more attention in the design?**
   a. *Which principle and why?*

IMPROVEMENT STRATEGIES

8. **How do you think we can further improve the workshop?**
9. **What do you think is important for the implementation of this workshop?**
   a. *What are supportive and/or challenging factors?*
10. **What else would you like to bring to the table?**

WRAP-UP

Wrap-up interview

“Do you have any further questions about this research?”

Right to adapt, supplement or delete

“If you have any adaptions or supplements, or would like us to leave out specific information, please let us know.”

Follow-up

“You will not receive a transcript or summary of the interview anymore. We gladly inform you of any further developments in our research.”

Attachment 2 to Beuken JA, Bouwmans ME, Dolmas DH, Hoven MF, Verstegen DM. *Qualitative expert evaluation of an educational intervention outline aimed at developing a shared understanding of cross-border healthcare.* GMS J Med Educ. 2024;41(2):Doc17. DOI: 10.3205/zma001672
